# Stockwell transform and semi-supervised feature selection from deep features for classification of BCI signals

**DOI:** 10.1038/s41598-022-15813-3

**Published:** 2022-07-11

**Authors:** Sahar Salimpour, Hashem Kalbkhani, Saeed Seyyedi, Vahid Solouk

**Affiliations:** 1 Faculty of Electrical and Computer Engineering, University of Tabriz, Tabriz, Iran; 2 Faculty of Electrical Engineering, Urmia University of Technology, Urmia, Iran; 3 University of California San Francisco and Berkeley, Berkeley, USA; 4 Department of IT and Computer Engineering, Urmia University of Technology, Urmia, Iran

**Keywords:** Biomedical engineering, Electrical and electronic engineering

## Abstract

Over the past few years, the processing of motor imagery (MI) electroencephalography (EEG) signals has been attracted for developing brain-computer interface (BCI) applications, since feature extraction and classification of these signals are extremely difficult due to the inherent complexity and tendency to artifact properties of them. The BCI systems can provide a direct interaction pathway/channel between the brain and a peripheral device, hence the MI EEG-based BCI systems seem crucial to control external devices for patients suffering from motor disabilities. The current study presents a semi-supervised model based on three-stage feature extraction and machine learning algorithms for MI EEG signal classification in order to improve the classification accuracy with smaller number of deep features for distinguishing right- and left-hand MI tasks. Stockwell transform is employed at the first phase of the proposed feature extraction method to generate two-dimensional time–frequency maps (TFMs) from one-dimensional EEG signals. Next, the convolutional neural network (CNN) is applied to find deep feature sets from TFMs. Then, the semi-supervised discriminant analysis (SDA) is utilized to minimize the number of descriptors. Finally, the performance of five classifiers, including support vector machine, discriminant analysis, *k*-nearest neighbor, decision tree, random forest, and the fusion of them are compared. The hyperparameters of SDA and mentioned classifiers are optimized by Bayesian optimization to maximize the accuracy. The presented model is validated using BCI competition II dataset III and BCI competition IV dataset 2b. The performance metrics of the proposed method indicate its efficiency for classifying MI EEG signals.

## Introduction

Brain–computer interface (BCI) is a powerful emerging technology that turns brain activity into helpful computer codes to drive mechanical devices for severely disabled people and patients with movement disorders^1^. BCI systems can restore, complete, replace, or rehabilitate human functions by incorporating brain activity in a low-cost and low-risk way without any muscle interference. Aside from healthcare and medical applications, BCI systems have contributed to manifold domains such as intelligent environment, advertisement, computer games, and education^2^. Classification of movement imagination signals is among the most significant contributions of the BCI systems in neurological rehabilitation. Due to noninvasive, high time resolution, proportionately simple operation, and low-cost, electroencephalogram (EEG) signal recorded from the scalp has been widely used in the BCI system in the fields of rehabilitation and reinforcement tools^3–5^.

There are some widely used EEG signals in BCI applications like steady-state visual evoked potential (SSVEP)^6^, which are brain reactions to visual stimuli at some particular frequencies, and slow cortical potential (SCP)^7^, that are more associated with movement functions. Also, evoked potential P300^8^ signal that has been commonly used as spellers, and motor imagery (MI)^9^. Recently, there are several studies on the use of brain activity over the sensorimotor regions by MI EEG signals, in which users imagine specific limb movements without really moving that part of the body to control the system. Since MI EEG signals can be collected easily and inexpensively, it has been employed for various applications such as controlling quadcopters, robots, electric wheelchairs, and other external devices^10,11^. Hence, to control a mechanical device the chief requirement is classification of brain activity patterns and translating those patterns into commands. While BCI systems have greatly improved, it is still challenging to accurately classify different MI states. Therefore, MI activity has been utilized for the BCI system in this work, with our goal to improve the classification performance with smaller number of features for MI tasks using three-step feature extraction technique.

Feature extraction and classification are the two salient factors in MI EEG signal processing. The analysis of the EEG signals begins with identifying their informative features. Typical spatial pattern (CSP) and CSP-based methods are popular feature extraction techniques in various MI studies^12–14^. Authors in Ref.^15^ have used the filter bank CSP (FBCSP) algorithm along with the principle component analysis (PCA) to select and reduce features from EEG signals which then are classified by the eXtreme gradient boosting (XGBoost) algorithm. Also, there have been several studies that use graph theory and functional connectivity to analyze EEG signals in MI tasks^16^. In another study, a frequency-based approach using CSP features from overlapping sub-bands was proposed for MI classification. Using all available channels, the method selects the most discriminating filter banks^17^. A number of studies have also examined the effectiveness of time-domain, frequency-domain, and the fusion of both information on the performance of MI EEG classification^18^. Recently, RNN-based metaheuristic algorithms, time-varying equations are applied to the control of robotic^19^, where an artificial dynamic system based on EMG signals and joint information was introduced to detect human motion intention in lower body parts. Also, neural network models have been used for and time-varying optimization problems^20^. Using a combination of RNN and CNN architectures, the work in Ref.^21^ classified a four-class MI on the BCI competition IV dataset 2a with the goal of having a model that could be applied to all participants. However, the performance of current studies in MI-EEG classification is still not comparable to other fields like image and speech recognition. The short-time Fourier transform (STFT) and the wavelet transform are also popular time–frequency approaches, which have been developed to extract the various EEG frequency characteristics over time^12,22,23^. In another reported study^24^, the STFT features of the input signals were extracted and then classified using a network based on ResNet. However, the limited width of the window in STFT results in constant resolution in both time and frequency domains; hence, it cannot provide proper frequency resolution at low frequency and good time resolution at high frequency. Several studies indicated that continuous wavelet transform (CWT) with variant mother wavelets represents appropriate multi-scale analysis for extracting significant features in the time–frequency resolution over MI EEG signals in BCI tasks^25–27^. Various machine learning methods have been employed to classify MI EEG signals, such as support vector machine (SVM)^28^, linear discriminant analysis (LDA)^29^, *k*-nearest neighbor (*k*NN)^30^, and other methods^23,31^. Deep learning models such as convolutional neural networks (CNNs) have been recently used in the BCI studies^32–34^.

In Ref.^27^, the authors considered the CWT and a four-layer CNN for classification. They improved average classification accuracy using three mother wavelets compared to the STFT on BCI competition II dataset III and BCI competition IV dataset 2b. In Ref.^33^, different mother wavelets were presented for time–frequency mapping of the EEG signals. Then a two-layer CNN was developed to classify a combination of TFMs of C3, Cz, and C4 channels into the left- and right-hand MI tasks. The accuracy rate of their work was 92.75% in dataset III from BCI competition II. Kant et al.^34^ converted the EEG signals into two-dimensional TFMs using the CWT. They used dataset III of BCI competition II in three different frequency spectrums and several transfer learning methods, including VGG19, AlexNet, VGG16, ResNet50, GoogleNet, and ResNet101, were applied to classify the MI data. They achieved maximum accuracy of 95.71% in full-band (8–30 Hz) by VGG19. Furthermore, time–frequency images obtained by Morlet wavelet transforms in Ref.^35^, were classified using an extended CNN with convolutional block attention modules (CBAM) with an accuracy of 90.7% on the BCI dataset III. The disadvantages of wavelet transform as the feature extraction method in these works are poor time resolution at low frequencies and finding an optimum window function before operation. Stockwell transform was presented to overcome the drawbacks of wavelet transform^36,37^. In Ref.^38^, Stockwell transform divided different MI signals into distinct frequency regions to prepare a distinguished feature vector combined with the CSP technique as a multi-step feature extraction method. The performances of three different classification techniques of least square-SVM (LS-SVM), random forest (RF), and artificial neural network (ANN) were compared. Accordingly, 95.55% accuracy was achieved with the LS-SVM classifier on BCI competition III dataset IIIa.

MI tasks have been classified with several different techniques, but currently, there is no superior algorithm that provides better results for most applications. Instead of using an individual classifier, the ensembles of different base classifiers have shown promising results for BCI^39,40^. Clearly, the quality of an ensemble method can be defined by its accuracy and diversity^41^. In Ref.^40^, a comparative study of three ensemble architectures based on three base machine learning classifiers of *k*NN, SVM, and Naive Bayes (NB) were represented to classify different feature sets extracted from MI data best performance was reported using Adaboost ensemble learning with multiple base classifiers. In Ref.^42^, a majority voting ensemble model of five individual classifiers [LDA, *k*NN, SVM, NB, and decision tree (DT)] showed a better average classification accuracy than every single classifier for multi-class motor imagery EEG signals. Although different ensemble learning methods can enhance the overall accuracy, they cannot consistently outperform the best individual classifier for some applications due to the different characteristics of the input datasets^43^.

BCI employs the brain activity for communication of paralyzed people with intact brain functions. However, the non-stationarity nature of brain activity and physiological artifacts contained in brain activity limit the performance and reliability of BCI technologies. Hence, our aim in this is to enhance the performance of MI task classification. Due to the nonlinear characteristics of MI EEG signals, it is preferable to employ time–frequency transforms to analyze these signals. Considering the explanations provided in the literature review, our objective is to improve the classification performance of BCI tasks using a smaller number of deep features and fusion algorithms before deep feature extraction and in decision levels. This paper uses the Stockwell transform to obtain the TFMs of MI EEG signals. Then, CNN is considered to elicit the robust deep features from TFMs. Since too many features have been extracted, they should be reduced to alleviate the computational complexity. To this end, we consider the semi-supervised discriminant analysis (SDA), which maximizes the separation of classes and estimates the basic geometric structure of the data. The selected CNN-based features are used as inputs for the five various machine learning classifiers. Finally, all these classifiers' performances and their combination are compared to find the most efficient method based on kappa values and classification accuracy.

This paper continues as follows. “Materials and methods” explains the dataset information and proposed methodology. The results of the performance assessment are given in “Results and discussion”. Finally, “Conclusion” concludes the paper.

## Materials and methods

**Figure 1:**
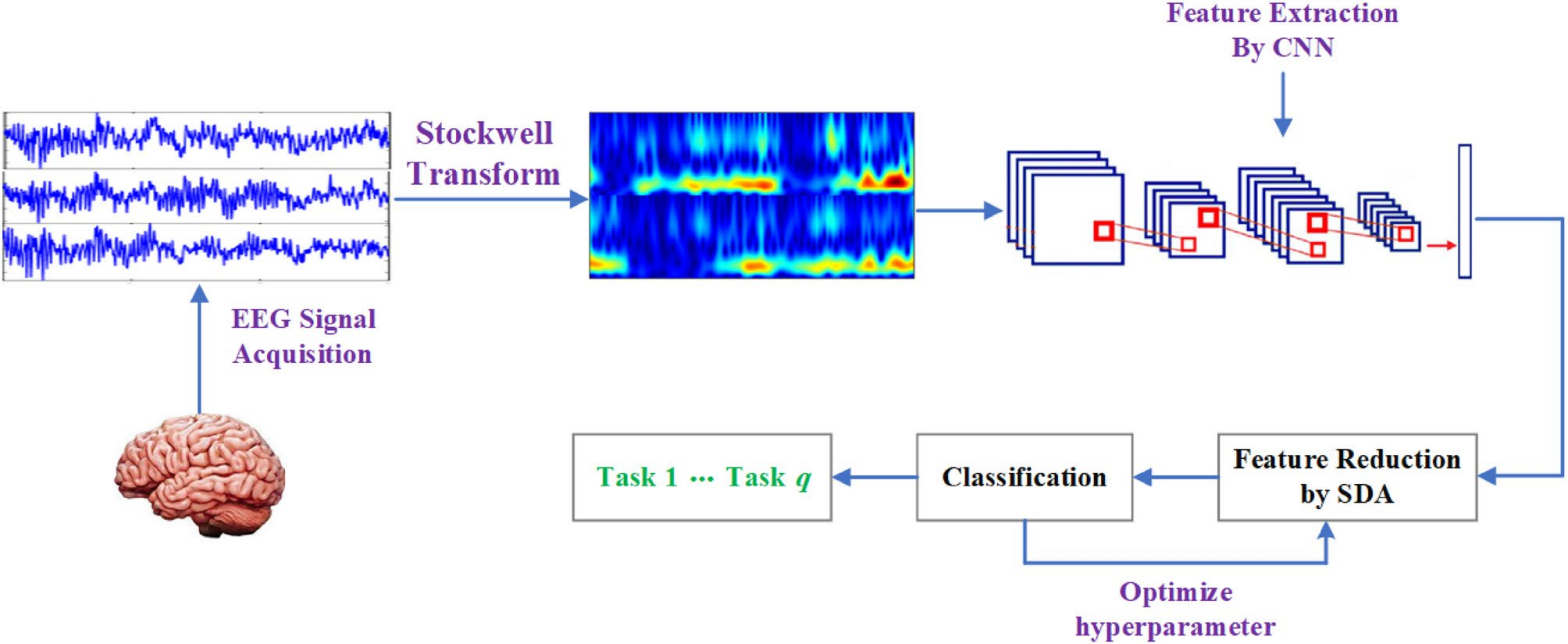
Block diagram of the proposed method for MI EEG classification.

Here, we explain the proposed method for MI EEG signal classification. In Fig. 1, the proposed method is shown in block diagram form. The proposed method generally consists of four steps, including (1) time–frequency analysis, (2) feature extraction, (3) feature reduction, and (4) classification. In the following, each step will be explained in detail.

### Dataset

The EEG signals for this study was taken from two datasets namely BCI competition II dataset III^44^ and BCI competition IV dataset 2b which respectivey refered as II–III and IV-2b^45^. Table 1 summarizes the detail of the datasets. In the following, a detailed description of each dataset will be presented.

**Table 1:** Summary of datasets used in this paper.

Dataset	No. of subjects	No. of categories	No. of channels	Sampling frequency (Hz)
II–III	1	2	3	128
IV-2b	9	2	3	250

**Figure 2:**
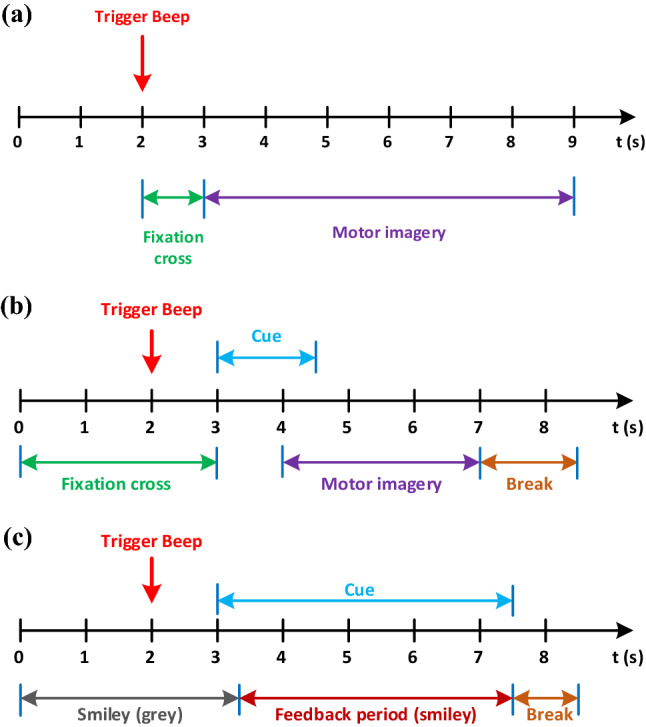
Timing scheme for recording EEG signals in each trial. (**a**) dataset II–III, (**b**) first two sessions of dataset IV-2b, (**c**) last three sessions of dataset IV-2b.

**Figure 3:**
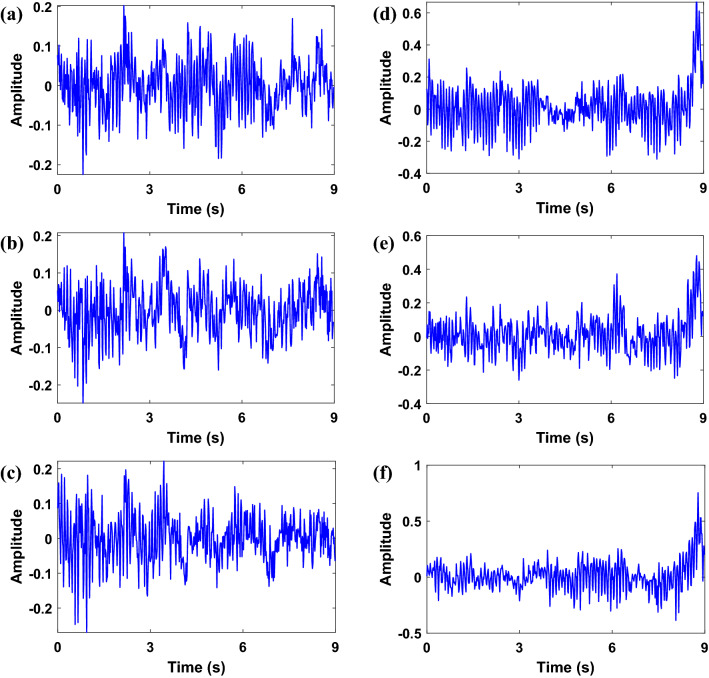
EEG signals from dataset II–III recorded during different tasks. (**a**) C3 channel of left-hand motion, (**b**) C4 channel of left-hand motion, and (**c**) Cz channel of left-hand motion, (**d**) C3 channel of right-hand motion, (**e**) C4 channel of right-hand motion, (**f**) Cz channel of right-hand motion.

The dataset II–III recorded the motor cortex's channels C3, C4 and Cz for a normal subject (a 25-year-old woman). It consists of MI task experiments for the left- and right-hand motions. In total, 280 trials of 9 s length are in the dataset. 140 of them are for training, and 140 are for testing. Following the first two seconds of silence, an acoustic stimulus was given at $t=2$ s, followed by the cross "+" display for one second. After that, a cue (left or right) was shown to the subject from $t=3$–9 s, and the subject was instructed to perform the imagery task. Each of the trials follows the same pattern as shown in Fig. 2a. The sampling rate was 128 Hz, and the signals were filtered between 0.5 and 30 Hz. Figure 3 presents one recording from each task in different channels.

The three-channel (C3, Cz and C4) EEG signals composing dataset IV-2b were collected from nine subjects^45^ under the sampling frequency of 250 Hz. To eliminate the signal noise, a band-pass filter in the range [0.5, 100] Hz is employed. Similar to the dataset II–III, imaginations of left hand movement and right hand movement were perfomred. EEG signals for each subjectwere recorded in five sessions, without feedback in the first two sessions, with feedback in the remaining three sessions. Each trail was recorded as shown in Fig. 2b–c.

### Time–frequency analysis

It should be mentioned that Motor movements, which are called ERS and ERD in brain activity, occur in the alpha (8–13 Hz) and beta (14–28 Hz) frequency bands, so we considered the output of Stockwell transform in the range 7–30 Hz. Hence, it is not required to remove the effect of the 50 Hz industrial frequency signal from raw EEG signal before computing Stockwell transform. Since EEG signals have nonlinear and non-stationarity characteristics, various time–frequency decomposition methods, such as STFT, wavelet transform, and Stockwell transform, have been conventionally used to analyze them. Due to the fixed window width in the STFT, the proper time and frequency resolution cannot be achieved simultaneously. The wavelet transform was proposed to overcome the problems related to Fourier transform by decomposing data into several scales, and each scale represents a particular resolution of the signals. The drawbacks of wavelet transform are choosing the optimum mother wavelet and losing the absolute phase of the data.

The Stockwell transform presented by Stockwell et al.^46^ is an extension of CWT and STFT. As an effective and efficient time–frequency decomposition method, the Stockwell transform gives high-frequency resolution at low frequencies while obtaining high time resolution at high frequencies. Therefore, in this study, the Stockwell transform was applied to represent EEG signals in time–frequency. The Stockwell transform of a continuous time-domain signal $x(t)$ is represented as:

1 $${S}_{x}\left(t,f\right)=\mathrm{exp}\left(j2\pi ft\right){W}_{x}\left(t,d\right),$$

where $$j= \sqrt{-1}$$ and

2 $${W}_{x}\left(\tau ,d\right)= {\int }_{-\infty }^{\infty }x\left(t\right)\omega \left(t-\tau ,d\right)dt,$$

denotes the CWT of signal $x(t)$ and $\omega (t,f)$ defines the Gaussian mother wavelet as:

3 $$\omega \left(t,f\right)=\frac{|f|}{\sqrt{2\pi }}\mathrm{exp}\left(-\frac{{f}^{2}{t}^{2}}{2}\right)\mathrm{exp}(-j2\pi ft),$$

where the factor $d$ represents the inverse of frequency $f (d=1/f)$. Hence, the expression of the Stockwell transform of the continuous signal $x(t)$ is given as^46^:

4 $${S}_{x}\left(\tau ,d\right)= \frac{|f|}{\sqrt{2\pi }}{\int }_{-\infty }^{\infty }x(t)\mathrm{exp}\left(-\frac{{f}^{2}({\tau -t)}^{2}}{2}\right)\mathrm{exp}\left(-j2\pi ft\right)dt.$$

According to (3), the window width in Stockwell transform depends on the frequency $f$. Thus, it becomes wider as the frequency decreases, and when the frequency increases, it becomes narrower^47^.

Let assume $x(nT)$, $n=0, 1, \dots , N-1$ be a discrete-time signal Acquired by sampling the continuous signal $x(t)$ where *T* is the sampling period. The discrete Stockwell transform is derived from the discrete Fourier transform (DFT) of the input signal. The *N*-point DFT of the signal can be expressed by

5 $$X\left\lfloor \frac{k}{NT} \right\rfloor = \frac{1}{N}\mathop \sum \limits_{n = 0}^{N - 1} x\left( {nT} \right)\exp \left( { - \frac{2j\pi kn}{N}} \right); \quad k = 0, 1, \ldots , N - 1.$$

Stockwell transform is defined in discrete form as being the projection of a vector onto a spanning set^46^. Discretization of (4) results in the discrete Stockwell transform:

6 $$S\left\lfloor {mT,\frac{n}{NT}} \right\rfloor = \mathop \sum \limits_{k = 0}^{N - 1} X\left\lfloor {\frac{k + n}{{NT}}} \right\rfloor G\left( {n,k} \right)\exp \left( { - \frac{2j\pi km}{N}} \right),$$

where $G\left(n,k\right)=\mathrm{exp}\left(-\frac{2{\pi }^{2}{k}^{2}}{{n}^{2}}\right)$ represents a Gaussian function and $n,m=0, 1, \dots , N-1$. The amplitude of Stockwell transform is needed for feature extraction, which is calculated as:

7 $$\left|S\right|= \sqrt{{(ReS\})}^{2}+{(ImS\})}^{2}}.$$

**Figure 4:**
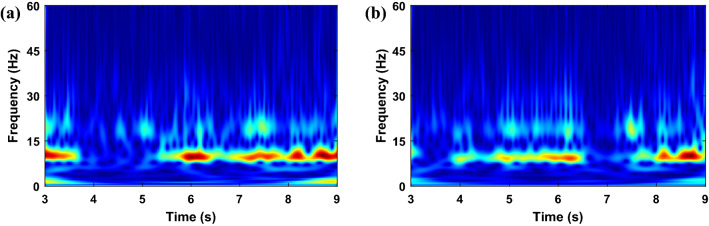
Absolute of Stockwell TFM corresponding to the left-hand MI signal from dataset II–III. (**a**) C3 channel, (**b**) C4 channel.

**Figure 5:**
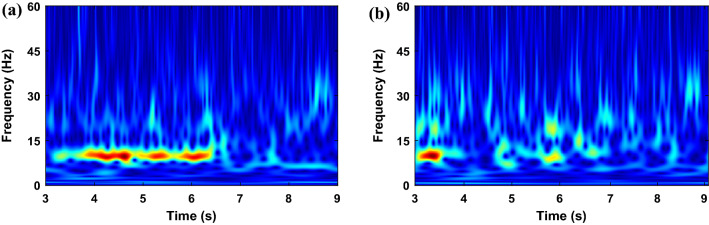
Absolute of Stockwell TFM corresponding to the right-hand MI signal from dataset II–III. (**a**) C3 channel, and (**b**) C4 channel.

**Figure 6:**
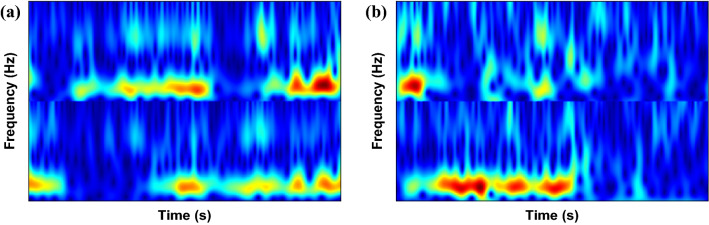
Demonstration of stacking TFMs from dataset II-III. (**a**) right-hand, and (**b**) left-hand.

It was demonstrated in Ref.^33^ that two electrodes placed in C3 and C4 are sufficient for classifying different imagery tasks. Hence, in this paper, the Stockwell transform was performed on signals obtained from C3 and C4 channels, and the corresponding absolute TFMs are shown in Figs. 4 and 5 for the left- and right-hand motions, respectively. The performing or even the imagination of motor movements can arouse specific patterns called event-related synchronization (ERS) and event-related desynchronization (ERD) in the brain activity, which occurs in the alpha (8–13 Hz) and beta (14–28 Hz) frequency ranges^48,49^. Since these phenomena are important in classifying MI EEG signals, a band-pass filter was applied on the raw EEG signals in 7–30 Hz. The TFMs of C3 and C4 electrodes in the range 7–30 Hz are then stacked vertically as shown in Fig. 6. As observed that TFMs of the left-hand and right-hand task are different, we can use them to classify MI tasks.

### Deep feature extraction by CNN

CNN is a network of deep neural connections designed for features extraction, classification, recognition, and detection applications. In this study, we utilize a CNN to extract deep features from TFMs. Each layer of CNN comprises two main building blocks; convolutional and pooling layers. The input of the CNN is stacked TFMs, and its output is a deep feature vector. The convolution layer is the first layer in CNN to extract features from an input TFM by applying different filters (kernels) and passing results to the pooling layer. Limiting the number of layers and the relevant parameters according to the number of training samples is an appropriate solution to avoid over-fitting and reduce the complexity of the functions^33^.

A mini-batch normalization layer and an activation layer are added after each convolution layer. The main objective of using a batch normalization layer between the convolutional layers is to normalize the outputs of each layer to have zero mean and unit variance, which can accelerate and improve the performance of deep neural networks^50^. The nonlinear activation function introduces nonlinearity to the neural network. There are several kinds of activation functions, and the most used ones are sigmoid, tangent hyperbolic, and rectified linear unit (ReLu) function^33^. ReLu is the most effective and popular activation function, which is defined as:

8 $$f\left(x\right)=\mathrm{max}\left(0,x\right).$$

Hence, for negative input, the output is equal to 0, and for positive input, it is a linear function. The ReLu function is faster and more straightforward than the previous two. As well as due to considerable variation in the outputs for positive inputs, it prevents the vanishing gradient problem. Accordingly, the ReLu activation function is chosen as the activation layer for the CNN in this paper. The pooling layer is the next layer, which is also called the sub-sampling or down-sampling layer. Max pooling and average pooling are the general pooling functions reducing the dimensions of the data by taking the maximum and the average value in the sampling area.

**Figure 7:**
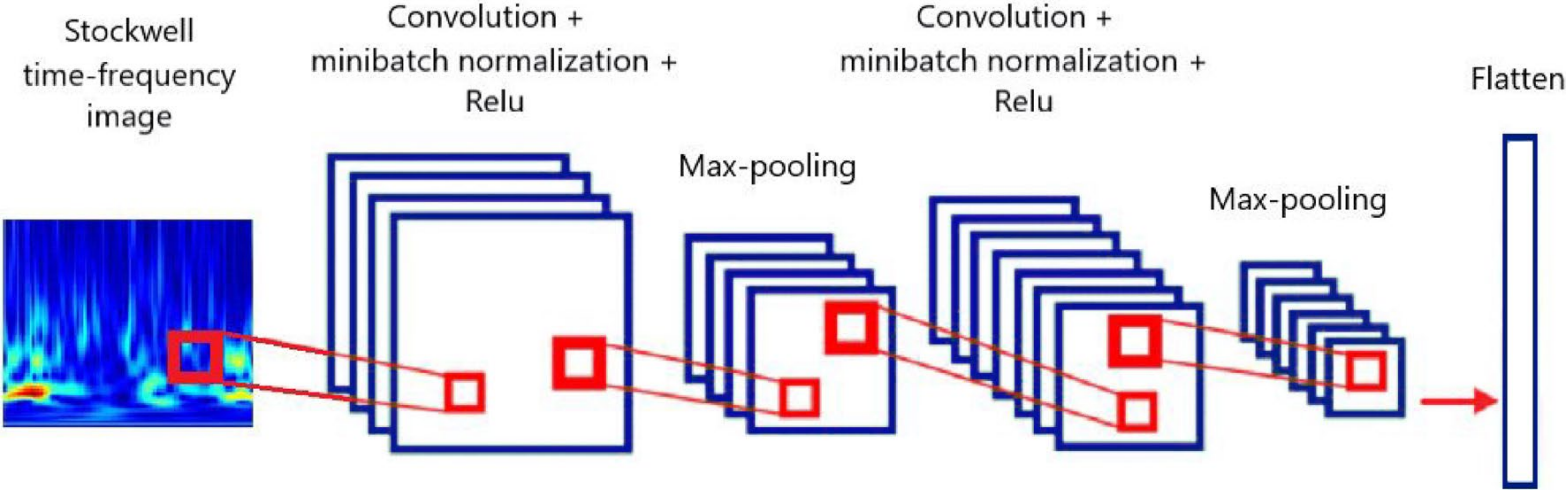
The structure of CNN with two convolutional layers.

In this research, CNN with two and three layers are considered to extract deep features from TFMs, where the first and second convolutional layers have eight and 16 kernels, respectively, and the last layer in three-layer CNN has 32 filters. The size of all filters is 3 × 3. The structure of the two-Layer CNN is depicted in Fig. 7.

Another approach to extract deeper features from images is using the pre-trained networks and adjusting their weights for new tasks. There are several pre-trained models for image recognition tasks, such as AlexNet, VGG16, VGG19^51^, Inception^52^, MobileNet^53^, and ResNet50^54^. In this paper, we extract features from the last pooling layer of the pre-trained AlexNet and the second fully connected layers of pre-trained VGG19 models and report their performance in our proposed model.

### Feature reduction

After deep feature extraction, the input TFM is represented by a vector with high-dimension. The several features maybe not be informative and have a higher correlation with each other. To select the most significant features and decrease the dimension of the feature vector, SDA is employed. SDA considers both labeled and unlabeled samples^55^. The labeled data points maximize the separation between different classes, while the unknown data estimate the basic geometric structure. A smooth discriminant function is fitted to the distribution of data by SDA.

Suppose that ${x}_{1},{x}_{2}, \dots ,{x}_{N} \epsilon {\mathbb{R}}^{L}$ denote the *N* training samples in *L*-dimensional space that correspond to *c* classes. The supervised version of SDA, i.e., linear discriminant analysis (LDA), only considers the labeled sample. LDA has the following objective function:

9 $${a}_{opt}=\underset{{\varvec{a}}}{\mathrm{argmax}}\frac{{{\varvec{a}}}^{T}{S}_{b}{\varvec{a}}}{{{\varvec{a}}}^{T}{S}_{w}{\varvec{a}}},$$

where ${S}_{w}$ and ${S}_{b}$ refer to the intra- and inter-class scatter matrices, successively, which are computed as follows:

10 $${S}_{b}={\sum }_{k=1}^{c}{N}_{k}\left({\mu }^{(k)}-{\varvec{\mu}}\right){\left({\mu }^{(k)}-{\varvec{\mu}}\right)}^{T},$$

11 $${S}_{w}={\sum }_{k=1}^{c}\left({\sum }_{i=1}^{{N}_{k}}\left({x}_{i}^{(k)}-{\mu }^{(k)}\right){\left({x}_{i}^{(k)}-{\mu }^{(k)}\right)}^{T}\right),$$

where ${N}_{k}$ denotes the number of training samples for *k*th class, ${\varvec{\mu}}$ is the total sample mean vector, ${\mu }^{(k)}$ is the mean vector of class *k*, and ${x}_{i}^{(k)}$ is the sample $i$ in class $k$. By defining the total scatter matrix ${S}_{t}={\sum }_{i=1}^{N}\left({x}_{i}-\mu \right){\left({x}_{i}-\mu \right)}^{T},$ we have ${S}_{t}={S}_{b}+{S}_{w}$. Thus, the objective function equals:

12 $${{\varvec{a}}}_{opt}=\underset{{\varvec{a}}}{\mathrm{argmax}}\frac{{{\varvec{a}}}^{T}{S}_{b}{\varvec{a}}}{{{\varvec{a}}}^{T}{S}_{t}{\varvec{a}}}.$$

If enough training samples are not available, overfitting may occur. Regularizers are typically used to prevent overfitting. In this case, the optimization problem is as follows:

13 $$\mathrm{max}\frac{{{\varvec{a}}}^{T}{S}_{b}{\varvec{a}}}{{{\varvec{a}}}^{T}{S}_{t}{\varvec{a}}+\beta J({\varvec{a}})},$$

where $J({\varvec{a}})$ determines the learning complexity of the hypothesis family, and the regulation coefficient *β* controls the balance between complexity of the model and the empirical loss. Considering natural regularizer, we have:

14 $$\begin{aligned} J\left( a \right) & = \mathop \sum \limits_{ij} \left( {{\varvec{a}}^{T} x_{i} - {\varvec{a}}^{T} x_{j} } \right)^{2} S_{ij} \\ & = 2\mathop \sum \limits_{i} {\varvec{a}}^{T} x_{i} D_{ii} x_{i}^{T} {\varvec{a}} - 2\mathop \sum \limits_{ij} {\varvec{a}}^{T} x_{i} S_{ij} x_{j}^{T} {\varvec{a}} \\ & = 2{\varvec{a}}^{T} X\left( {D - S} \right)X^{T} a \\ & = 2{\varvec{a}}^{T} XLX^{T} a, \\ \end{aligned}$$

where *S* is the weight matrix defined as:

15 $${S_{ij}} = \left\{ {\begin{array}{ll} {1,}&{{\rm{if}}\,{x_i} \in {N_p}\left( {{x_j}} \right)\,\,{\rm{or}}\,\,{x_j} \in {N_p}\left( {{x_i}} \right)}\\ {0,}&{{\rm{otherwise,}}} \end{array}} \right.$$

where ${N}_{p}({x}_{i})$ stands for the set of *p* nearest neighbors of ${x}_{i}$. *D* is a diagonal matrix; its entries are column (or row, since *S* is symmetric) sum of *S*, ${D}_{ii}={\sum }_{j}{S}_{ij}$ and $L=D-S$ is the Laplacian matrix. Hence, the objective function of SDA can be formulated as:

16 $$\underset{{\varvec{a}}}{\mathrm{max}}\frac{{{\varvec{a}}}^{T}{S}_{b}{\varvec{a}}}{{{\varvec{a}}}^{T}\left({S}_{t}+\beta XL{X}^{T}\right){\varvec{a}}}.$$

The objective function is maximized by the projective vector a which is defined by the maximum eigenvalue solution to the generalized eigenvalue problem:

17 $${S}_{b}{\varvec{a}}=\lambda \left({S}_{t}+\boldsymbol{\alpha }XL{X}^{T}\right){\varvec{a}}.$$

Considering $A=\left[{a}_{1},{a}_{2},\dots ,{a}_{c}\right]$, where *c* is the number of non-zero eigenvalues, samples are embedded as:

18 $$x\to z={A}^{T}x.$$

As observed the performance of SDA depends on the regulation parameter *β*. In this paper, the Bayesian optimization is employed to find the optimum value of the parameter *β* which yields in the highest classification accuracy.

### Classification

In this paper, five well-known machine learning classifiers were applied to classify two-class feature vectors, and their results are compared. Due to different behavior of classifiers in some cases, a fusion method was used to enhance the reliability of overall classification accuracy by combining the decisions of classifiers.

#### Support vector machine (SVM)

Vapnik^56^ introduced the SVM as the robust classifier. Due to its lower computational complexity and easy processing of small datasets, it has been commonly employed in various BCI studies^4,57–59^. The optimal hyperplane in SVM maximizes the marginal distance between classes. In this paper, linear SVM was considered.

#### Discriminant analysis

Low computation requirement and easy implementation make discriminant analysis one of ideal classifiers for EEG based-BCIs^29,60^. In the discriminant analysis method, the boundary among classes is defined based on maximizing the ratio of inter-class variance and minimizing intra-class variance. The discriminant analysis classification technique uses Bayes’ Theorem to predict which class the test data belongs to^61^.

#### k-Nearest neighbor (kNN)

The *k*NN approach is a famous statistical method in machine learning-based classification algorithms. The *k*NN is a simple classifier in MI tasks^59,62^ classifies each test data by considering the k distance metrics between the test data and those of the closest classes in the feature space. As a result, the parameter *k* is an essential key in the performance of the *k*NN.

#### Decision tree (DT)

DT is a supervised machine learning technique in which a dataset is continuously split into subsets based on a particular parameter. This classifier uses a tree-like structure that contains the root, internal decision, and terminal nodes. The root node is considered as the whole dataset sorted into branches. The intermediate subsets are called decision nodes, and the terminal node shows the predicted classes^63^.

#### Random forest (RF)

The RF is a supervised machine learning classifier proposed by Leo Breiman in 2001^64^. RF classifiers collect decisions of multiple DT classifiers where a random subset of the features is selected to train each DT classifier. This process increases the variation among the trees; hence it overcomes overfitting. Eventually, combining the results of all DTs determines the final decision on new data.

#### Ensemble of classifiers

**Figure 8:**
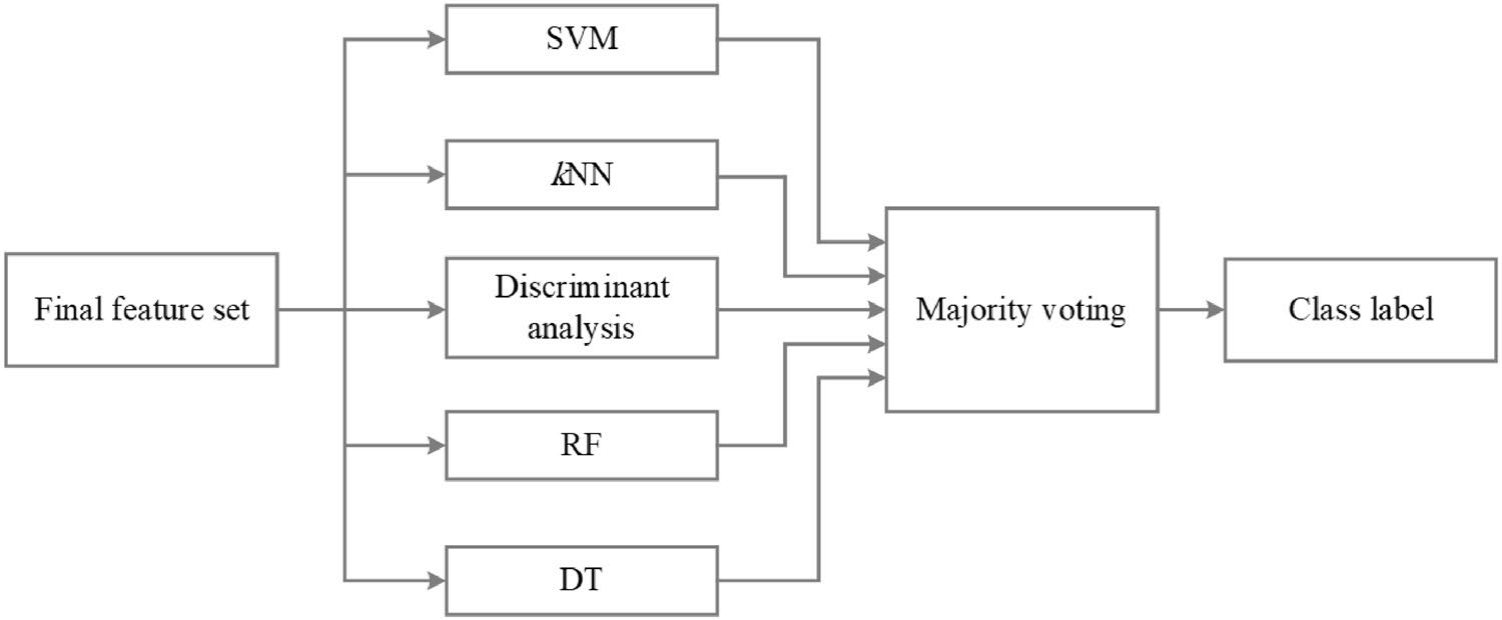
Ensemble of five classifiers (decision-level fusion) to classify the features ontained from SDA.

The ensemble is the combination of two or more individual classification models to improve the overall performance. A robust ensemble model is based on two essential parameters: the accuracy and diversity of classifiers^41^. In this research, the majority voting ensemble, one of the most popular combination approaches for classification^65^, was used to combine the results of five classifiers for the final decision, as shown in Fig. 8. In this model, the final class prediction is the one that receives more than half of the votes among the base classifiers.

### Computational complexity

The proposed method consists of three main parts including feature extraction, feature reduction and classification. The time complexities of computing Stockwell transform and feature extraction using CNN are $O(N)$ and $O({N}_{s})$, respectively, where $N$ is the number of samples of EEG signal and ${N}_{s}$ in the number of pixels in input TFM^46,66^. Similar to LDA, the computational complexity of SDA is $O({N}_{tr}{d}_{i}^{2})$, where ${N}_{tr}$ is the number of training samples and ${d}_{i}$ is the dimension of input feature vector^55^. Finally, the computational complexities of SVM^67^, *k*NN^68^, decision tree^69^, and random forest^70^ classifiers are $O({N}_{tr}^{3})$, $O({N}_{tr}k{d}_{r})$, $O({d}_{r}{N}_{tr}{\mathrm{log}}_{2}{N}_{tr})$ and $O(TD)$, respectively, where ${d}_{r}$ is the dimension of reduced feature vectors, $T$ is the size of forest and $D$ denotes the maximum depth.

### Informed consent

All methods were carried out in accordance with relevant guidelines and regulations and were approved department of medical informatics, institute for biomedical engineering, university of technology, Graz, Austria.

## Results and discussion

This section reports the results of the conducted experiments. The performance of the proposed model was evaluated through classification accuracy, kappa score, confusion matrix, precision, and sensitivity. The classification accuracy as the most widely used measure defined as^34^:

19 $$Acc.=\frac{TP+TN}{TP+FP+TN+FN}\times 100,$$

where TP (true positive) is the number of correctly classified feature sets, and TN (true negative) is the number of correctly rejected ones. FN (false negative) is the number of feature sets identified wrongly, and FP (false positive) is the number of wrongly rejected feature sets. The values for all these parameters are derived from the confusion matrix. Sensitivity, also known as recall, is the ability of the model to predict all the true positives of each specific class. It is obtained as^71^:

20 $$\text{Sens.}=\frac{TP}{TP+FN}\times 100.$$

The precision reflects the proportion of accurate positive predictions out of the total number of samples classified as positive:

21 $$\text{Prec.}=\frac{TP}{TP+FP}\times 100.$$

Besides, the kappa score was applied to measure the classification performance of the proposed model and eliminate the randomness effects^72^. It is calculated as follows:

22 $$kappa=\frac{(Acc-rAcc)}{(1-rAcc)},$$

where *rAcc.* denotes the random accuracy, which is defined as

23 $$rAcc.=\frac{1}{{N}_{c}},$$

where *N*_*c*_ is the number of the classes, which equals two in the considered dataset.

### Data preparation

**Figure 9:**
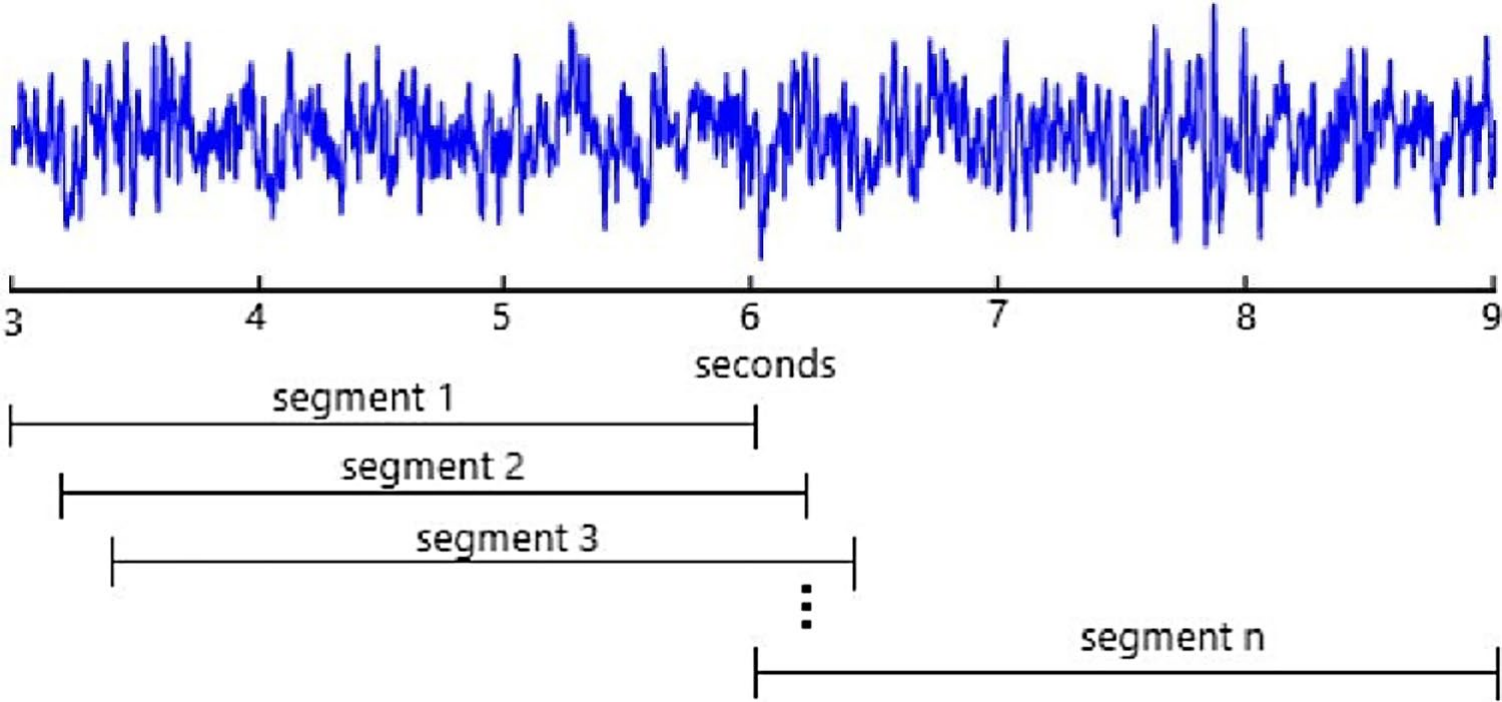
Demonstration of three-second segments of dataset II–III with a stride of 250 ms.

Each raw EEG signal of dataset II–III has a duration of nine seconds. However, the last six seconds of the original EEG signal are considered for MI classification. We consider the six-second duration of the trial and multiple smaller segments within the trial. The objective of sliding time windows within the trial is to discover the most effective time duration in classification accuracy. In this work, three windows with the length of two, three, and four seconds were considered to extract EEG segments from both training and test datasets with a stride of 250 ms. The first segments start from the third second of the original signal, and the last ones finished at the trial end. As an example, segments with three seconds time duration are shown in Fig. 9. The 50% of data was used for training and the remaining data was considered for test phase.

The first three sessions of dataset IV-2b were considered in this paper. The MI segment in this dataset has the length of three seconds. Hence, we only consider the two-second sliding windows with a stride of 250 ms. The 50% of data was used for training and the remaining data was considered for test phase.

### Feature reduction

**Figure 10:**
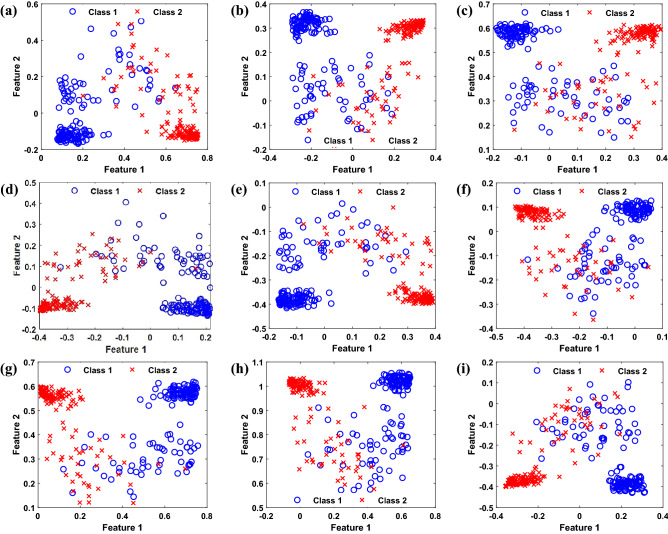
Features obtained from SDA considering the different length and location for the sliding window. (**a**) second window of length two seconds, (**b**) seventh window of length two seconds, (**c**) 12th window of length two seconds (**d**) second window of length three seconds, (**e**) seventh window of length 3 s, (**f**) 12th window of length three seconds (**g**) second window of length three seconds, (**h**) sixth window of length three seconds, and (**i**) ninth window of length three seconds.

The CNN automatically extracts the high-dimensional deep features from each TFM. All extracted features are not informative, and most of them are redundant. As mentioned, SDA is considered for feature reduction. The size of input feature vector depends on the structure of CNN which equals to 48,400 for proposed two-layer CNN. According to characteristics of SDA, the size of the reduced feature vector equals to the number of classes which is equal to two in this paper. In simulations, 2/3 of training samples are considered labeled data, and the remaining ones are treated as unlabeled data. Simulations show that there are two non-zero eigenvalues; hence, SDA reduces the number of features to two, which reduces the computational complexity considerably. The scatter plot of the features generated by SDA for different lengths and locations of the sliding window is shown in Fig. 10. It is observed that the length of the sliding window and its location has a considerable effect on the distribution of features generated by SDA. Hence, classification accuracy is expected to vary by length and location of the prediction window, shown in the following.

### Results of whole MI trials

We considered the optimization procedure to find the hyperparameters of classifiers. For SVM classifier, the box constraint and kernel type, i.e., linear, quadratic, cubic, or gaussian, are found by Bayesian optimization. In addition, for gaussian kernel, its scale was also optimized. In the case of *k*NN classifier, number of neighbors, distance metric and distance weight were obtained by Bayesian optimization. Distance metric is chosen from Euclidean, Mahalanobis, cubic and cosine. The weighting scheme is also chosen from equal, inverse, and squared inverse. For decision tree, the maximum number of splits is found by Bayesian optimizer. Gini's diversity index was considered as split criterion and a node in a tree is height-balanced if the heights of its subtrees differ by no more than one. The discriminant type of discriminant classifierwas found among linear, quadratic, diagonal linear, and diagonal quadratic by grid search. Finally, Bayesian optimizer finds the minimum leaf size and number of predictors to sample for random forest classifier.

A comparative study of the proposed model's classification accuracy and kappa score in Tables 2, 3, 4 and 5 for different classifiers. These tables compare the performance of five single classifiers and their fusion with the majority voting method based on deep features extracted by two- and three-layers CNN and pre-trained models, including AlexNet and VGG19. In order to evaluate the effectiveness of the Stockwell transform, the results of Stockwell TFM are compared with the Morlet wavelet transform and STFT, which showed relatively better results than other mother wavelets in recent studies^27,33,73^.

**Table 2:** Classification accuracy and Kappa scores for different machine learning approaches considering two- and three-layer CNN for Stockwell transform, Morlet wavelet transform and STFT on the dataset II–III.

Classifier	Stockwell transform				Morlet wavelet transform				STFT			
	Accuracy (%)		Kappa score		Accuracy (%)		Kappa score		Accuracy (%)		Kappa score	
	2-layer	3-layer	2-layer	3-layer	2-layer	3-layer	2-layer	3-layer	2-layer	3-layer	2-layer	3-layer
SVM	97.14	91.42	0.943	0.828	94.99	92.85	0.899	0.857	94.28	92.14	0.886	0.843
Discriminant	95.71	92.14	0.914	0.843	94.28	91.42	0.886	0.828	94.99	91.42	0.899	0.828
*k*NN	96.43	92.85	0.929	0.857	94.99	90.71	0.899	0.814	94.28	89.99	0.886	0.799
DT	89.28	89.99	0.786	0.799	89.99	90.71	0.799	0.814	90.71	89.28	0.814	0.786
RF	94.28	92.14	0.886	0.843	93.56	91.42	0.871	0.828	94.28	90.71	0.886	0.814
Majority voting	97.14	91.42	0.943	0.828	94.28	92.85	0.886	0.857	94.99	91.42	0.899	0.828

**Table 3:** Classification accuracy and Kappa scores for different machine learning approaches considering two- and three-layer CNN for Stockwell transform, Morlet wavelet transform and STFT on the dataset IV-2b.

Classifier	Stockwell transform				Morlet wavelet transform				STFT			
	Accuracy (%)		Kappa score		Accuracy (%)		Kappa score		Accuracy (%)		Kappa score	
	2-layer	3-layer	2-layer	3-layer	2-layer	3-layer	2-layer	3-layer	2-layer	3-layer	2-layer	3-layer
SVM	85.05	78.73	0.701	0.574	79.32	75.77	0.586	0.515	77.87	74.73	0.557	0.495
Discriminant	77.68	75.09	0.554	0.502	74.50	72.86	0.490	0.457	73.95	73.45	0.479	0.469
*k*NN	78.32	73.27	0.566	0.465	73.73	70.68	0.475	0.414	69.46	68.96	0.389	0.379
DT	82.41	77.59	0.648	0.552	73.55	71.64	0.471	0.433	70.59	70.27	0.412	0.405
RF	82.27	75.82	0.645	0.516	78.77	74.91	0.575	0.498	76.59	74.73	0.532	0.495
Majority voting	86.05	81.36	0.721	0.627	79.77	75.95	0.595	0.519	76.86	74.81	0.537	0.496

**Table 4:** Classification accuracy and Kappa scores for different machine learning approaches considering AlexNet and VGG19 networks for Stockwell transform, Morlet wavelet transform and STFT of dataset II–III.

Classifier	Stockwell transform				Morlet wavelet transform				STFT			
	Accuracy (%)		Kappa score		Accuracy (%)		Kappa score		Accuracy (%)		Kappa score	
	Alex net	VGG 19	Alex net	VGG 19	Alex net	VGG 19	Alex net	VGG 19	Alex net	VGG 19	Alex net	VGG 19
SVM	91.42	92.85	0.828	0.857	94.99	90.71	0.899	0.814	91.42	89.99	0.828	0.799
Discriminant	90.71	91.42	0.814	0.828	94.28	89.28	0.886	0.786	89.99	89.28	0.799	0.786
*k*NN	92.14	92.14	0.843	0.843	94.28	90.01	0.886	0.800	91.43	88.57	0.8286	0.771
DT	89.99	84.23	0.799	0.685	93.56	89.29	0.871	0.786	89.99	89.29	0.799	0.786
RF	91.42	92.85	0.828	0.857	94.28	88.57	0.886	0.771	91.43	89.29	0.828	0.786
Majority voting	92.14	92.85	0.843	0.857	94.28	89.28	0.886	0.786	92.13	89.99	0.843	0.799

**Table 5:** Classification accuracy and Kappa scores for different machine learning approaches considering AlexNet and VGG19 networks for Stockwell transform, Morlet wavelet transform and STFT of dataset IV-2b.

Classifier	Stockwell transform				Morlet wavelet transform				STFT			
	Accuracy (%)		Kappa score		Accuracy (%)		Kappa score		Accuracy (%)		Kappa score	
	Alex net	VGG 19	Alex net	VGG 19	Alex net	VGG 19	Alex net	VGG 19	Alex net	VGG 19	Alex net	VGG 19
SVM	81.59	77.82	0.632	0.556	76.55	74.63	0.531	0.493	77.09	73.14	0.542	0.463
Discriminant	75.46	74.46	0.509	0.489	72.95	72.36	0.459	0.447	72.54	71.81	0.451	0.436
*k*NN	78.86	70.09	0.577	0.402	72.46	68.77	0.449	0.375	67.23	67.91	0.345	0.358
DT	74.18	77.14	0.484	0.543	71.37	70.78	0.427	0.416	69.41	70.22	0.388	0.404
RF	77.99	73.41	0.559	0.468	76.41	74.73	0.528	0.495	74.23	73.81	0.485	0.476
Majority voting	83.37	78.91	0.667	0.578	78.09	73.54	0.562	0.471	75.59	73.96	0.512	0.479

Table 4 shows that the Morlet wavelet transform has a better average classification accuracy than the Stockwell transform when the pre-trained AlexNet network is applied for extracting deep features. However, the maximum achieved accuracy is still less than the best achieved accuracy using Stockwell transform by other deep CNN models. Most classifiers have achieved comparatively better performance with proposed Stockwell-based features in the classification of EEG signals. The results indicate that in the proposed model, for the dataset II–III, the majority voting classifier has the highest classification accuracy of 97.14% and 86.05%, respectively on datasets II–III and IV-2b, with Stockwell transform using two-layer CNN. In general, two-layer CNN has the highest classification accuracy based on the Stockwell transform. The results show that, although the fusion model obtained better accuracies in most cases, it does not always give the best classification results. Regarding kappa scores, the proposed method has the maximum value of 0.943 and 0.721, respectively on datasets II–III and IV-2b, for using Stockwell transform, while Morlet wavelet transform and STFT resulted in lower kappa values.

Table 6 presents the confusion matrix, sensitivity, and precision for our proposed fusion model based on the Stockwell transform related to two-layer CNN. It demonstrates the correspondence between the predicted and actual labels for each action class in the considered datasets. As observed, the model's sensitivity for right-hand imagery movements achieved the better rate than that of the left hand.

**Table 6:** Confusion matrix for fusion model.

			Predicted labels		Precision (%)	Sensitivity (%)
			Left hand (%)	Right hand (%)		
Actual labels	II–III	Left hand	97.86	2.14	99.28	97.86
		Right hand	0.71	99.29	97.89	99.29
	IV-2b	Left hand	84.82	15.18	86.95	84.82
		Right hand	12.73	87.27	87.27	87.27

### Classification results of sliding window

Here we discuss the location of the sliding windows on the accuracy of the proposed method. Tables 7, 8 and 9 present the performances of classification methods on three different segments size using CNN with two layers for dataset II-III. Regarding two-second segments, the best accuracy of 98.57% was obtained by *k*NN and majority voting in 3.75–5.75 s time duration, and the segments extracted from the last two seconds of the trial showed the lowest accuracy rate. Similarly, the results in Table 8 indicate that the SVM, *k*NN, and majority voting classification algorithms have attained the highest accuracy and kappa value of 99.29% and 0.986, respectively, in the 3.25–6.25 s time duration. In contrast, the lowest accuracy has been mainly achieved for the last segment. Table 9 shows similar results for four-second segments with the highest classification accuracy of 98.57% by the SVM and majority voting classifiers, while the DT classifier reported the minimum amounts in all segments.

**Table 7:** Classification accuracy and kappa score results for two-second segments using Stockwell transform for datset II–III.

Classification method	Best			Worst			Mean	
	Accuracy (%)	Kappa score	Duration (s)	Accuracy (%)	Kappa score	Duration (s)	Accuracy (%)	Kappa score
SVM	97.86	0.957	3.75–5.75	85.72	0.714	6.75–8.75	93.36	0.867
Discriminant	97.86	0.957	3.75–5.75	85.72	0.714	6.75–8.75	92.77	0.855
*k*NN	98.57	0.971	3.75–5.75	86.43	0.728	6.50–8.50	93.57	0.871
DT	96.43	0.929	4–6	84.99	0.699	7.00–9.00	91.01	0.821
RF	97.86	0.957	4–6	85.72	0.714	7.00–9.00	92.65	0.853
Majority voting	98.57	0.971	3.75–5.75	85.72	0.714	6.50–8.50	93.32	0.866

**Table 8:** Classification accuracy and kappa score results for three-second segments using Stockwell transform for datset II–III.

Classification method	Best			Worst			Mean	
	Accuracy (%)	Kappa score	Duration (s)	Accuracy (%)	Kappa score	Duration (s)	Accuracy (%)	Kappa score
SVM	99.29	0.986	3.25–6.25	89.29	0.786	6.00–9.00	93.96	0.879
Discriminant	98.57	0.971	3.25–6.25	88.57	0.771	6.00–9.00	93.68	0.874
*k*NN	99.29	0.986	3.25–6.25	89.29	0.786	6.00–9.00	93.68	0.874
DT	95.71	0.914	3–6	85.71	0.714	5.50–8.50	91.09	0.822
RF	98.57	0.971	3–6	89.29	0.786	6.00–9.00	92.97	0.859
Majority voting	99.29	0.986	3.25–6.25	90.71	0.814	6.00–9.00	93.68	0.874

**Table 9:** Classification accuracy and kappa score results for four-second segments using Stockwell transform for datset II–III.

Classification method	Best			Worst			Mean	
	Accuracy (%)	Kappa score	Duration (s)	Accuracy (%)	Kappa score	Duration (s)	Accuracy (%)	Kappa score
SVM	98.57	0.971	3.25–7.25	91.42	0.828	5.00–9.00	94.36	0.887
Discriminant	97.85	0.957	3.25–7.25	91.42	0.828	5.00–9.00	94.28	0.886
*k*NN	97.85	0.957	3.25–7.25	90.71	0.814	5.00–9.00	94.68	0.894
DT	95.01	0.901	3–7	87.14	0.743	5.00–9.00	91.27	0.825
RF	97.14	0.943	3–7	90.01	0.801	5.00–9.00	93.96	0.879
Majority voting	98.57	0.971	3.25–7.25	90.71	0.814	5.00–9.00	94.91	0.898

Since the length of MI segment in dataset IV-2b is three seconds, we only considered the windows with the length of two seconds. Table 10 summarizes the best, worst and mean accuracies for considered classifiers. It is observed that majority voting achieves the highest accuracy of 89.02% considering the window between 3.25 and 5.25 s. The worst accuracy of 63.59 belongs to the DT and discriminant classifiers in the range 4–6 s. Also, majority voting classifier has the highest average accuracy as 80.78%.

**Table 10:** Classification accuracy and kappa score results for two-second segments using Stockwell transform for datset IV-2b.

Classification method	Best			Worst			Mean	
	Accuracy (%)	Kappa score	Duration (s)	Accuracy (%)	Kappa score	Duration (s)	Accuracy (%)	Kappa score
SVM	88.05	0.761	3.25–5.25	69.51	0.391	4–6	80.56	0.611
Discriminant	81.68	0.634	3–5	69.51	0.391	4–6	77.46	0.549
*k*NN	81.32	0.626	3–5	69.95	0.399	3.75–5.75	76.55	0.531
DT	87.55	0.751	3.25–5.25	63.59	0.272	4–6	75.59	0.512
RF	84.45	0.689	3.5–5.5	64.23	0.285	4–6	75.17	0.503
Majority voting	89.02	0.781	3.25–5.25	69.95	0.399	3.75–5.75	80.78	0.616

**Figure 11:**
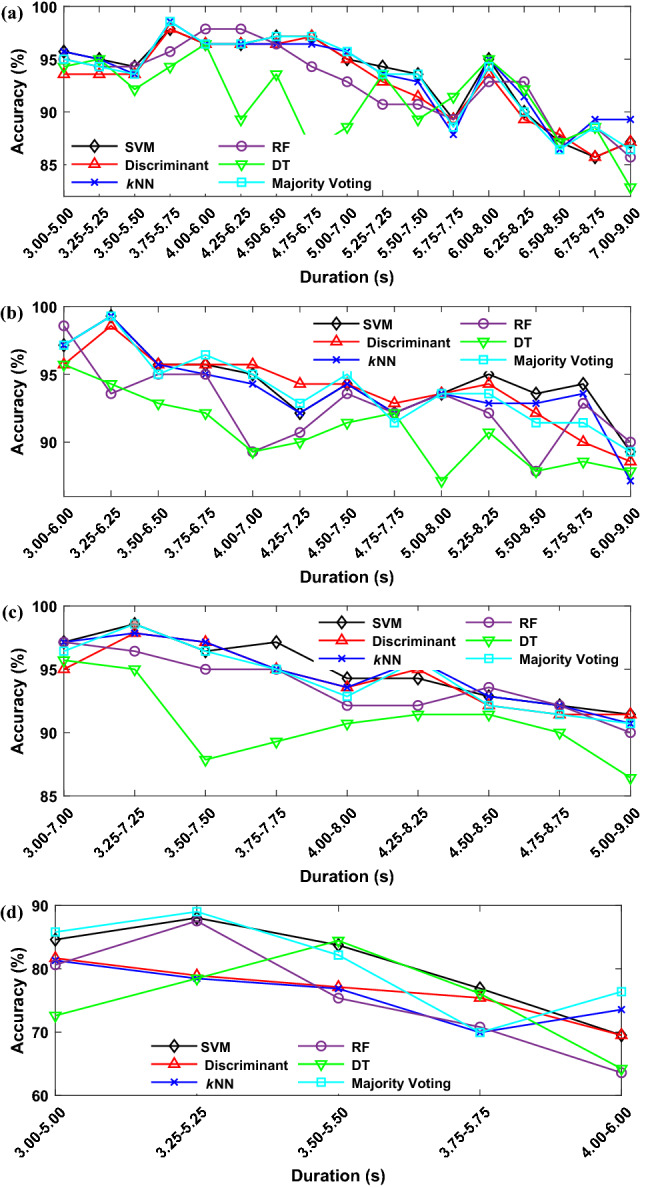
Effect of the location of sliding window on the classification accuracy. (**a**) two-second sliding window for dataset II–III (**b**) three-second sliding window for dataset II–III (**c**) four-second sliding window for dataset II–III, **(d)** two-second sliding window for dataset IV-2b.

Figure 11a depicts the classification accuracy of the classifiers on two-second segments. The results demonstrate that all classification methods have performed comparatively better in classification accuracy and kappa value in the beginning seconds of the MI task. Then, the overall classification accuracy is trending downward, where the lowest performance has been yielded in the last segments. A similar trend can be seen in Fig. 11b,c for three-second and four-second time duration segments, respectively. Also, Fig. 11d presents the accuracies of different two-second sliding windows for dataset IV-2b. Therefore, finding the most effective time duration of the signals depends on various factors such as the segment size, the delay in conducting the imagery task according to the cue, and the subject's concentration during the trial.

Another finding of this study is that although the majority voting ensemble improves the classification performance in some segments, a minor improvement was observed in the overall accuracy compared with individual classifiers, especially SVM. Therefore, it can be concluded that using a simple machine learning algorithm such as SVM as the final classification method in the proposed model is better than applying the fusion model in terms of accuracy, processing time, and computational complexity.

The confusion matrix of the best-achieved classification accuracy of 99.29% by SVM, *k*NN and majority voting classifier, which is for 3.25–6.25 s time duration, is given in Table 11. For the dataset IV-2b, the maximum accuracy of 89.02% in the duration of 3.25–5.25 is obtained by majority voting classifier. The overall results demonstrate the efficiency of the proposed model at classifying MI EEG signals.

**Table 11:** Confusion matrix, sensitivity, and precision for best accuracy.

			Predicted labels		Precision (%)	Sensitivity (%)
			Left hand (%)	Right hand (%)		
Actual labels	II–III	Left hand	98.57	1.43	100	98.57
		Right hand	0	100	98.59	100
	IV-2b	Left hand	88.28	11.82	89.74	88.28
		Right hand	10.09	89.91	88.38	89.91

### Effect of feature reduction on accuracy

Here, we evalute the effect of feature reduction on the accuracy of the proposed method. To this end, we compare the performance of the proposed method with other feature reduction schemes such as PCA, locality preserving projection (LPP)^74^, and neighborhood preserving embedding (NPE)^75^. We also presented the accuracy considering the original feature vector. The results are given in Table 12. The results indicate that the SDA considerably enhances the accuracy of classification.

**Table 12:** Accuracy of different feature reduction schemes.

Method		Proposed method (%)	Proposed method without feature reduction (%)	PCA (%)	LPP (%)	NPE (%)
Accuracy	II–III	99.29	86.42	90.71	87.85	91.43
	IV-2b	89.02	76.36	81.18	78.54	82.27

### Performance comparison

Various approaches have been proposed to classify MI signals. In order to compare the classification results of BCI competition II dataset III, the best result achieved in this study is compared with other methods found in the existing studies in terms of accuracy (Table 13). The authors in Ref.^76^ have proposed STFT-based TFM as input and considered a single layer CNN, stacked autoencoders (SAE), and a combination of them (CNN-SAE) to classify MI EEG signals. They reported classification accuracy of 90% using CNN-SAE on BCI competition II dataset III. In Ref.^33^, a two-layer CNN was developed to classify a combination of TFMs of C3, Cz and C4 channels using different mother wavelets. The best accuracy rate of their work for the current dataset was 92.75% based on the 3.25–6.25 s time duration. In Ref.^77^ extracted spatial–temporal features using the multivariate empirical mode decomposition were classified with SVM and achieved 85.2%. Also, higher-order dynamic mode decomposition and multichannel singular spectrum decomposition hybridization were considered in Ref.^78^ for feature extraction. The authors in Ref.^27^ utilized three various mother wavelets, i.e., Morlet, Bump, and Mexican wavelets, to extract the TFMs. They achieved better classification accuracy using the Bump wavelet for combined mu and beta bands and a one-dimensional CNN as the classification method. In Ref.^29^, a flexible analytic wavelet transform (FAWT) was implemented to decompose MI EEG signals into multiple sub-bands. Then, the reduced statistical features by the multidimensional scaling (MDS) technique were classified using the LDA classifier. The model resulted in 94.29% classification accuracy on dataset II–III.

**Table 13:** Performance comparison of various studies.

Study	Year	Dataset	Method	Accuracy (%)
Tabar et al.^76^	2016	II–III	STFT with deep learning	90
Xu et al.^33^	2018		TFM of the wavelet transform, one layer CNN	92.75
Lee et al.^27^	2019		TFM of the wavelet transform, two-layer CNN	92.9
You et al.^29^	2020		Flexible analytic wavelet transform, LDA	94.29
Kim et al.^73^	2020		Magnitude and phase of TFM, CNN	94.6
Kant et al.^34^	2020		CWT filter-bank, Transfer learning	95.71
Zhand et al.^24^	2021		STFT, dynamic multi-scale ResNet	90.47
Chen et al.^35^	2021		Time–frequency image subtraction, CBAM-CNN	90.7
Huang et al.^18^	2021		Dual-stream CNN	90.71
Proposed model	–		CNN-based features from Stockwell TFM, feature selection by SDA, SVM	99.29
Lu et al.^79^	2016	IV-2b	Deep learning based on restricted boltzmann machines	84.2
Hernández-González et al.^80^	2021		Spectrograms + scalograms, CNN + LSTM	73.8
Degdevir et al.^81^	2021		Hjorth algorithm, ANOA, SVM	82.58
Malan et al. ^82^	2022		Dual-tree complex wavelet, NCA, SVM	84.02
Han et al.^83^	2022		STFT, parallel CNN	83.0
Proposed model	–		CNN-based features from Stockwell TFM, feature selection by SDA, SVM	89.02

In Ref.^73^, the magnitude and phase information extracted from CWT images' real and imaginary parts were fed to a one-layer CNN. The proposed method achieved the best 94.6% classification accuracy. The method described in Ref.^34^ explored various transfer learning models such as VGG19, AlexNet, VGG16, SqueezeNet, ResNet50, GoogleNet, DenseNet201, ResNet18, and ResNet101 to classify Morse wavelet-based TFMs. The method reached up to 95.71% classification accuracy in the case of VGG19. In Ref.^24^, a new dynamic multi-scale layer was added to the ResNet network to extract the multi-scale characteristics from the STFT features of the input signal. They have obtained an accuracy of 90.47%. The authors in Ref.^35^, employed two CBAMs in a two-layer CNN for classification of the subtraction TFMs of two C3 and C4 channels. Huang and colleagues in Ref.^18^ developed a dual-stream convolutional neural network based on AlexNet and achieved the highest accuracy of 90.71% by combining time and frequency information.

In the following, the some papers focused on dataset IV-2b are reviewed. Boltzman machines were employed in Ref.^79^ and reaches the accuracy of 84.2%. A combination of spectrogram and scalogram as input TFM given to CNN + LSTM structure was yielded the accuracy of 73.8%^80^. The combination of Hjorth parameters as extracted features, ANOVA for feature selection and SVM for classification reaches the accuracy of 82.58% in Ref.^81^. Dual-tree complex wavelet was used in Ref.^82^ to extract the time–frequency component of EEG signals. After selection of efficient features by NCA, the SVM classified the BCI MI EEG signals which yields the accuracy of 84.02%. In Ref.^83^, parallel CNNs were used classify of TFM obtained from STFT and the accuracy of 83% was achieved. The results show that the proposed method with the accuracy of 89.02% outperforms the recently introduced methods.

Most of the mentioned works have incorporated wavelet transform-based approaches to extract the feature of the whole-time duration of the MI EEG signals. While, in the present study, finding the location and duration of the most exciting part of the signal has been investigated in detail, and better accuracy and kappa value have been yielded by the Stockwell transform-based features.

## Conclusion

In this paper, a new approach based on Stockwell TFMs of EEG signals was proposed to enhance the classification accuracy and reduce the deep features to classify the left- and right-hand movement imagery. In this study, the Stockwell transform was to decompose the time–frequency content of EEG signals, since it provides better resolution than the others such as wavelet transform and STFT. We considered early fusion scheme and combined the Stockwell transform of different channels before deep feature extraction. Compared to other studies which mainly focused on one specific scheme for the classification stage, we examined different machine learning methods as well as their fusion to cover each other's weaknesses. Four CNN models were used to extract high-dimensional deep features, where the TFMs of C3 and C4 channels in the frequency range of [10 30] Hz were concatenated and considered as input of CNN. Since there are a large number of features extracted by CNN, SDA was employed to reduce them to two. The classification accuracy of different optimized classifiers and a fusion of them by the majority voting method were compared. The whole MI EEG signals with six seconds length and multiple small segments of the signal with the lengths of two, three, and four seconds with different locations were considered for classification. Results indicate that the fusion model does not outperform the maximum individual classifier performance in most cases. The accuracy of 99.29% and 89.02% were obtained for datasets II–III and IV-2b, respectively, by two-layer CNN. The accuracy achieved in this study demonstrates the efficiency of our proposed method in comparison with previous studies on BCI competition II dataset III. Hence, the proposed method can be used in BCI systems to provide reliable communication between paralyzed people and external devices. Results also indicated that most information of EEG signals is at the beginning EEG samples of MI task, and there is less information at the last EEG samples of MI task.

Considering the single-modal, i.e., EEG, for feature extraction, can limit the performance of the proposed scheme when there are more than two classes. Also, training process of CNN takes long time which is dependent to the structure of CNN. In order to enhance the performance of classification, especially in the case multi-class scenarios, the multimodal scheme, such as combination of functional near-infrared spectroscopy (fNIRS) and EEG can be considered. Also, considering attention-based deep structures can further increase the classification accuracy. In order to further reduce the complexity of the proposed scheme, the effect of each layer on the accuracy can be analyzed by employing explainable artificial intelligence.
